# Does pre-spawning catch and release angling affect offspring telomere dynamics in Atlantic salmon?

**DOI:** 10.1093/conphys/coad018

**Published:** 2023-04-24

**Authors:** Eleanor Duncan, Magdalene Papatheodoulou, Neil B Metcalfe, Darryl McLennan

**Affiliations:** School of Biodiversity, One Health and Veterinary Medicine, Graham Kerr Building, University of Glasgow, G12 8QQ Glasgow, UK; School of Biodiversity, One Health and Veterinary Medicine, Graham Kerr Building, University of Glasgow, G12 8QQ Glasgow, UK; School of Biodiversity, One Health and Veterinary Medicine, Graham Kerr Building, University of Glasgow, G12 8QQ Glasgow, UK; School of Biodiversity, One Health and Veterinary Medicine, Graham Kerr Building, University of Glasgow, G12 8QQ Glasgow, UK

**Keywords:** welfare, stress, senescence, fishing, fish, Conservation biology

## Abstract

The practice of ‘catch and release’ (C&R) angling confers a balance between animal welfare, conservation efforts and preserving the socio-economic interests of recreational angling. However, C&R angling can still cause exhaustion and physical injury, and often exposes the captured fish to the stress of air exposure. Therefore, the true conservation success of C&R angling depends on whether the angled individuals then survive to reproduction and whether there are any persisting effects on subsequent generations. Here we tested the hypothesis that the stress of C&R angling is then passed on to offspring. We experimentally manipulated the C&R experience of wild adult salmon prior to the spawning season. These parental fish either underwent a C&R simulation (which involved exercise with/without air exposure) or were left as control individuals. We then measured the telomere length of the arising offspring (at the larval stage of development) since previous studies have linked a shorter telomere length with reduced fitness/longevity and the rate of telomere loss is thought to be influenced by stress. Family-level telomere length was positively related to rate of growth. However, the telomere lengths of the salmon offspring were unrelated to the C&R experience of their parents. This may be due to there being no intergenerational effect of parental stress exposure on offspring telomeres, or to any potential effects being buffered by the significant telomere elongation mechanisms that are thought to occur during the embryonic and larval stages of development. While this may suggest that C&R angling has a minimal intergenerational effect on offspring fitness, there have been numerous other reports of negative C&R effects, therefore we should still be aiming to mitigate and refine such practices, in order to minimize their impacts on fish populations.

## Teaser

While #x2018;catch and release#x2019; (C#x0026;R) angling allows a better balance between angler interest and conservation effort, the overall experience can still be stressful. Here we experimentally test whether C#x0026;R stress experienced by parent fish is also passed on to the next generation (using offspring telomere length measurement as proxy).

## Introduction

Atlantic salmon populations are under increasing pressure from factors such as climate change (Todd *et al.,* 2008; Jonsson and Jonsson, 2009; Dadswell *et al.,* 2022), habitat degradation (Lawrence *et al.,* 2016; McLennan *et al.,* 2019; [Bibr ref23][Bibr ref23]), increased aquaculture intensity (Torrissen *et al.,* 2013; Gilbey *et al.,* 2021) and fishing pressure (Hard *et al.,* 2008; Saura *et al.,* 2010). The majority of wild salmon captures are now due to rod angling (e.g. in the UK; Marine Scotland Marine Scotland, 2021), which has traditionally involved keeping any captured individuals. However, one way of partly mitigating the detrimental effects of angling on salmon populations is to release the captured fish back into their natural environment afterwards, a protocol commonly referred to as ‘catch and release’ (C&R) angling. The practice of C&R angling confers a balance between conservation efforts and preserving the socio-economic interests of recreational angling. There has been a steady increase in C&R angling on a global scale (Van Leeuwen *et al.,* 2021). For example, in Scotland, it is thought that over 90% of the salmon captured by rod fisheries in 2020 were released back into the wild (Marine Scotland, 2021).

This rise in C&R angling is generally considered a success story. However, the conservation success of C&R angling depends on whether the angled individuals then survive to reproduce and whether there are any persisting effects on subsequent generations. Irrespective of whether an individual is released after capture, the general process of being angled can cause a multitude of negative effects on the fish. For example, C&R angling can still cause physical injury (e.g. through scale loss or by hook injury) and often still involves exposing the captured fish to air. For most fish species, a functional requirement of the gills is that the filaments be submerged in water. Therefore, exposing fish to air may cause gill filaments to collapse and/or adhere to each other, which can then limit gas exchange at the gill lamellae and cause acute hypoxic effects (Cook *et al.,* 2015). This can result in a range of physiological effects, such as reduced respiration, fluctuating heart rates and a build-up of carbon dioxide in the blood (Ferguson and Tufts, 1992; Cook *et al.,* 2015). In addition to injury and air exposure, fish captured by rod and line angling will often fight to exhaustion (Olsen *et al.,* 2010; Lennox, 2018). This has the potential to deplete already-limited energy reserves (due to salmon suspending feeding on return to their natal stream), which may also trigger stress hormone pathways (Killen *et al.,* 2003; Suski *et al.,* 2003; Donaldson *et al.,* 2014; Raby *et al.,* 2015). Therefore, while the mortality rate associated with C&R angling is thought be relatively low (Cook *et al.,* 2015; Twardek *et al.,* 2018; Smukall *et al.,* 2019), the overall capture experience may still initiate an acute physiological stress response (Wedemeyer and Wydoski, 2008; Smukall *et al.,* 2019). For example, experimental C&R studies have shown that heart rate and stress hormone levels can remain elevated for a significant amount of time after the fish has been released (Pankhurst and Dedualj, 1994; Anderson *et al.,* 1998). This in turn can cause immunosuppression, thus making fish more vulnerable to pathogens, such as the opportunistic fungi *Saprolegnia* spp (Papatheodoulou *et al.,* 2022).

Any stress initiated in these parental fish may then persist into subsequent generations (either directly or indirectly) via parental effects (Mileva *et al.,* 2011; Burton and Metcalfe, 2014; Redfern *et al.,* 2017), meaning that the offspring of C&R angled fish may in turn share the burden of the stress experienced by their parents. For example, experimental studies on Atlantic salmon have shown that C&R exposure can negatively impact a number of life history traits, such as a female’s total clutch size (Richard *et al.,* 2013; Papatheodoulou *et al.,* 2022). Bouchard *et al.* (2022) also found that Atlantic salmon that had undergone C&R angling had lower reproductive success (measured as the number of offspring surviving to 3 months) than non-caught salmon, but the study was unable to tell whether this was due to poorer parental survival, lower breeding success or lower offspring fitness and survival. In this regard, measuring the telomere lengths of the offspring could offer novel insights into potential intergenerational stress effects of C&R angling.

Telomeres occur in most eukaryotic species as repetitive short sequences of DNA at the ends of chromosomes that act as protective caps. This is partly due to the ‘end replication problem’ whereby linear DNA is unable to fully replicate during cell division (Chan and Blackburn, 2004). While this leads to a gradual reduction in the length of the telomeres as cells continue to divide, the presence of the telomeres prevents the loss of DNA sequence from the central coding regions of chromosomes (Shay and Wright, 2019). In addition to cell division, the rate of telomere attrition can also be accelerated by environmental stressors (Angelier *et al.,* 2018; Chatelain *et al.,* 2020). This is thought to be partly due to oxidative stress pathways (Reichert and Stier, 2017; Barnes *et al.,* 2019; Coluzzi *et al.,* 2019). For example, exposure to stressful environmental conditions can trigger complex physiological stress responses (via the hypothalamic–pituitary–adrenal or hypothalamic–pituitary–interrenal axis), which in turn can elevate the rate of aerobic metabolism and the production of reactive oxygen species (ROS) as by-product. If left unquenched, ROS may then cause oxidative damage to cellular structures including proteins, lipids and DNA (von Zglinicki, 2002; Reichert and Stier, 2017), and telomeres are thought to be particularly sensitive to oxidative damage due to their high guanine content (Haussmann and Marchetto, 2010; Monaghan and Ozanne, 2018). While telomere elongation mechanisms such as the expression of the enzyme telomerase may act to counterbalance this loss, such mechanisms are often downregulated in post-embryonic somatic tissues (Gomes *et al.,* 2010; Tian *et al.,* 2018). Therefore, in the absence of elongation mechanisms, telomeres may shorten to such an extent that the central coding region of a chromosome becomes vulnerable, which can then trigger the senescence or death of that cell (Victorelli and Passos, 2017; Zhu *et al.,* 2019). An accumulation of senescent cells is related to age-related deterioration (Kowald *et al.,* 2020), which has led to the suggested pathway between telomere length and biological state.

It is therefore unsurprising that a number of studies have identified links between environmental stress and the rate of telomere erosion (for reviews see Angelier *et al.,* 2018; Chatelain *et al.,* 2020). For example, Atlantic salmon juveniles were found to have shorter telomeres for a given body size when experimentally reared in a harsher environment (McLennan *et al.,* 2016). Any stress experienced by parents may also affect the telomere dynamics (i.e. telomere length and rate of loss) of the next generation via genetic and/or parental effects pathways (Tarry-Adkins *et al.,* 2009; Asghar *et al.,* 2015; Marasco *et al.,* 2019; Young *et al.,* 2022), which in turn may depend on offspring sex (Marasco *et al.,* 2019) and/or offspring environment (Young *et al.,* 2022). While the estimation of telomere heritability is complicated by the fact that telomere lengths change through life and are under the influence of extrinsic factors, a recent meta-analysis reported an average heritability score of ~ 45% (Chik *et al.,* 2022). Therefore, though complex, these abovementioned studies highlight how parental stress can alter the cellular physiology of offspring, thus also having potential consequences for the offspring’s rate of organismal ageing and longevity. Thus, any stress incurred by adult Atlantic salmon during a C&R angling event has the potential to carry over into the next generation and alter the telomere dynamics of offspring.

In this study, we experimentally manipulated the C&R experience of wild parental salmon towards the end of their return spawning migration. These parental fish either underwent a C&R simulation (which involved exercise with/without air exposure) or were left as control individuals that did not undergo any simulation. We then measured telomere length in the arising offspring (at the larval stage of development) to test the hypothesis that the experience of C&R angling leads to intergenerational stress effects on offspring telomere length. Testing this hypothesis will allow a greater understanding of how far-reaching the effects of this angling practice are in order to better mitigate any potential effects and better understand the conservation requirements of the Atlantic salmon.

## Methods

### Capture of parent fish/C&R protocol

All procedures carried out in this study were approved under UK Home Office Project License PB948DAAO. Wild parental fish were captured between November and December 2018 at the Loch na Croic fish trap (57° 60’N, 4°63’W) during their return spawning migration from the sea. These parental fish were then held in large circular holding tanks (diameter, 4 m; depth, 1.5 m; water flow, 60 l/min; maximum stocking density, 60 fish per tank), where they were kept in single-sex groups and given 24–48 h to recover. The water in these holding tanks was supplied directly from the River Blackwater, and the temperature (6 ± 1.5°C) was recorded on an hourly basis using a temperature data logger (HOBO Pendant Temperature/Light 64 K Data Logger, Onset Computer Co., USA).

**Figure 1:**
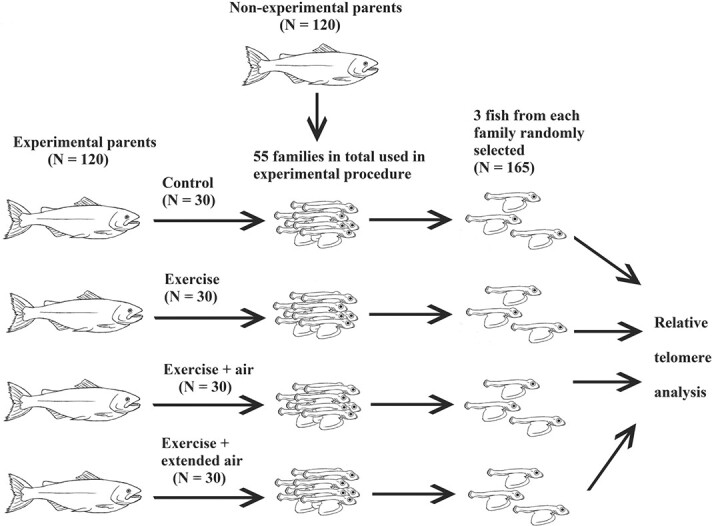
A visual overview of the experimental protocol**.** In total, 120 experimental families were created, whereby one of the parents (either male or female) was exposed to one of four experimental treatments, and then crossed with a random non-experimental fish. Seven families per treatment per parental sex were randomly selected and transferred to the aquarium facilities at the University of Glasgow (a total of 56 families). Of these, 55 families were used in this study for telomere measurement (3 fish per family, *N* = 165).

In total, 120 experimental families were created, whereby one of the parents (either male or female) was assigned to one of four experimental treatments, and then crossed with a random non-experimental fish using *in vitro* fertilizations (IVF); see Fig 1. The four experimental treatments were designed to represent realistic experiences of C&R angling and are similar in protocol to previous studies (Struthers *et al.,* 2018; [Bibr ref60][Bibr ref60]). The experimental treatments were as follows: (1) control, (2) exercise only, (3) exercise and air exposure (for 60 s) and (4) exercise and extended air exposure (for 120 s). The experimental parents were selected at random from the holding tanks; however, females were only used on the condition that they had not released the egg clutch into their body cavity and were therefore not yet in spawning condition (i.e. were not yet fully ripe). Each selected fish was anaesthetized (with clove oil 20 ppm) and measured for body mass (± 1.0 g) and body fork length (± 0.5 cm). Individuals were then randomly allocated to one of the four treatment groups using a number generator (random.org; version 2.0), before being tagged with an individually colour-coded Floy tag (indicating treatment) and a passive integrated transponder (PIT) tag (indicating individual) and were then allowed to recover for 24–48 h. In total, there were 15 fish per treatment per sex, thus creating 120 experimental families in total.

Experimental parent fish in the control treatment (#1) were not exposed to any kind of C&R simulation. Parents in the other three groups (#2, 3, 4) were all exercised individually in a separate large circular holding tank (diameter, 4 m; height, 1.5 m; water depth, 0.18 m) by manually chasing the fish (e.g. by lightly tapping the fish on its side and/or tail) for 210 s without rest, in order to simulate the level of exercise that a fish might experience during angling. In addition to this chase simulation, groups #3 and #4 also underwent an air exposure simulation (by being held in a knotless net) for either 60 s or 120 s respectively, with these times being based on observations of C&R angling (Lamansky and Meyer, 2016). Following treatment, each fish was then placed in one of two post-protocol recovery tanks (same dimensions as before, one tank per sex), where they then remained for a minimum of five days. After this point, IVF matings were conducted so that each experimental fish was then crossed with a non-experimental fish. The timing of these matings depended on when the females came into spawning condition (i.e. were fully ripe and had released their eggs into the abdominal cavity; identifiable by a soft belly). Prior to the stripping of gametes, parents were anaesthetized using a mild 20 ppm clove oil solution. Using standard hatchery procedures, each female was stripped of eggs that were then fertilized with sperm from a single male (that was either experimental or non-experimental, depending on the experimental status of the female).

### Rearing of offspring

The fertilized egg clutches were then held overwinter in family specific groups at the SSE hatchery in Contin, N. Scotland. The water in this hatchery is sourced from the River Blackwater, therefore the incubation temperatures were the same as in the wild. In the following spring (April 2019), the offspring had reached the larval ‘alevin’ stage of development, whereby the eggs have hatched but the free-moving embryos continue to be nourished from a yolk sac rather than exogenous food. At this point, seven families per treatment per parental sex were randomly selected and transferred to the aquarium facilities at the University of Glasgow (a total of 56 families). At the same time, a further six individuals from the same sub-selection of families were randomly selected and euthanized with an overdose of clove oil and then stored in 100% ethanol for subsequent telomere analysis. The siblings that were transferred to the University aquarium facilities were housed as separate family groups in 2 L compartments of a recirculating stream system (water flow = 0.988 L/s). These families were initially reared in darkness at 7°C until they had reached the end of the larval stage, i.e. the yolk sacs had been used up and they were ready to start feeding on exogenous food. The date of first feeding was recorded for each of the experimental families (i.e. for each 2 L compartment) based on the following criteria: (1) all individuals had visibly used up their yolk sacs, (2) a proportion of individuals were actively swimming in the water column and (3) at least 5 individuals within the group were actively responding to a provided food stimulus (i.e. were searching for and/or consuming the food). Additionally, the fork length of each fry within a family was measured (± 1 mm) on the day of first feeding. This was achieved by photographing each individual from above when held in a water-filled container (water depth = 1 cm) with a submerged reference, thus allowing fork length to be subsequently measured using ImageJ software. From the point of first feeding, conditions within the stream system were then gradually changed to a 12 L:12D photoperiod and 12°C over the course of two months (May–June). During this time, total fry mortality was recorded for each family, calculated as the sum of mortality during the first three months of feeding, and expressed as a percentage of the number of offspring on day 1 of feeding.

### Relative telomere length measurement in offspring

Three ethanol-preserved alevins (that had been sampled at the time of transfer to the University aquarium facilities) were randomly selected from each of the 56 experimental families. One family was found to not be properly preserved in ethanol; therefore, this family was not used (bringing the total number to 165 individual samples from 55 families). The entire caudal fin was carefully removed from each individual and then lysed overnight at 56°C in 180 μl buffer ATL + 20 μl of proteinase K solution (20 mg/ml). DNA was then extracted from these lysates using the DNeasy Blood and Tissue Kit (Qiagen), following the manufacturer’s protocol. DNA concentration and purity were measured spectrophotometrically using a Nanodrop 8000, which confirmed that all samples met the recommended A260/280 ratio and had a DNA concentration > 30 ng/μl.

The quantitative PCR method outlined by Cawthon (2002) was used to measure relative telomere length in all samples. Relative telomere length was calculated as a ratio (T/S) of telomere repeat copy number (T) to a single copy number gene (S). The primers ‘Tel1b’ and ‘Tel2b’ designed by Cawthon (2002) with modifications from Epel *et al.* (2004) were used for amplification of the telomere repeats. The single-copy gene used for the analysis was the Atlantic salmon recombination activating gene 1 (RAG-1), using the same primer sequences as in McLennan *et al.* (2021). The telomere assay thermal profile was as follows: 95°C for 15 minutes, followed by 27 cycles of 15 seconds at 95°C, then 30 seconds at 58°C and then 30 seconds at 72°C. This was then followed by a melt curve analysis, to confirm the amplification of a single PCR product. The thermal profile for the RAG-1 assay was as follows: 15 minutes at 95°C, followed by 40 cycles of 15 seconds at 95°C, 30 seconds at 60°C, and 30 seconds at 72°C. This was again followed by a melt curve analysis. The PCRs were performed using an Mx3005P qPCR system (Agilent) with each of the assays—telomere (T) and single copy gene (S)—carried out using separate 96 well plates, running each sample in triplicate for both assays. Additionally, every plate contained a seven-fold serial dilution (in triplicate) of a reference sample ranging from 0.625–40 ng/well, as well as a non-target control (NTC, also in triplicate). The serial dilution of DNA was created from a pool of DNA using all 165 individuals, and the NTCs were composed of all reagents but minus any DNA, in order to check for non-specific binding and potential contamination between sample wells. Each reaction contained 12.5 μl 2x ABsolute Blue qPCR SYBR Green Mix low ROX (Fisher Scientific), forward and reverse primers and DNA (wells containing sample, standard) or water (wells containing NTC) in a total volume of 25 μl. Both T and S assays were performed using 5 ng of DNA/well. Primer concentrations were 500 nM for the telomere assay (Tel1b and Tel2b) and 200 nM for the RAG-1 assay (SalmonRAG1-F and SalmonRAG1-R). The 165 samples were randomly distributed across seven sets of PCR plates (one T and one S plate per set) resulting in a total of 14 PCR plates.

qPCR data were analysed using qBASE software for windows (Hellemans *et al.,* 2007), as described in McLennan *et al.* (2016). The efficiency ranges of the telomere and RAG-1 assays were 92.1–108.0 and 90.9–101.3 respectively, with the qBASE software additionally helping to control for differences in amplification efficiency between targets and plates (assessed from the standard curve of each plate). The qBASE software also helped to correct for inter-run variation by using three points from the standard curve (40, 10 and 1.25 ng/well) as inter-run calibrators. The standard curve was also used to calculate an inter-assay coefficient of variation of the T/S ratios (which was 7.04). Out of the 165 focal individuals included in the qPCR analysis, telomere length was successfully measured for 164 individuals. One individual was excluded because the sample lay out with the acceptable limits of the RAG-1 single copy gene standard curve, even after having repeated the assay (from the DNA dilution step onwards).

### Statistical methods

Data are available via a Dryad Digital Repository (McLennan *et al.,* 2023). Using R (R version 4.1.1 in R Studio version 1.4.1717), we first ran a linear mixed effects model using the lme4 and LmerTest packages (Bates *et al.,* 2015; Kuznetsova *et al.,* 2017) to investigate whether the *Relative telomere length* of the offspring varied as a function of our two experimental manipulations. *Parental stress category* was included as the first explanatory variable to test whether offspring telomere length varied among the four parental stress categories: (1) control, (2) exercise only, (3) exercise and air exposure (60 s) and (4) exercise and extended air exposure (120 s). *Sex of experimental parent* was included as the second explanatory variable to test whether any potential inter-generational effect of C&R angling might in turn depend on whether it was the mother or the father that had experienced the C&R simulation. *Family ID* was included as a random effect in this mixed effect model to account for non-independence between siblings. The telomere data were log10 transformed, due to the typical non-Gaussian distribution of the antilog telomere data (Lin *et al.,* 2019). Significance in this and the subsequent analysis was determined at a threshold of *P* = 0.05.

In the second analysis we calculated the mean telomere length for each of the 55 experimental families (i.e. the mean of the three offspring) and then ran a general linear model (lm function) to test whether family level telomere length varied as a function of two key life history traits. *Fork length at first feeding* was included as the first explanatory variable in order to test for a correlation between family-level growth potential and telomere length; a link between growth rate and telomere length has previously been shown in several different life stages of juvenile salmon (McLennan *et al.,* 2016; McLennan *et al.,* 2021). *Offspring mortality rate* was included as the second explanatory variable to test whether family-level telomere length was linked to the overall mortality rate of that family, quantified as the percentage of mortality during the first three months of feeding.

## Results

**Figure 2:**
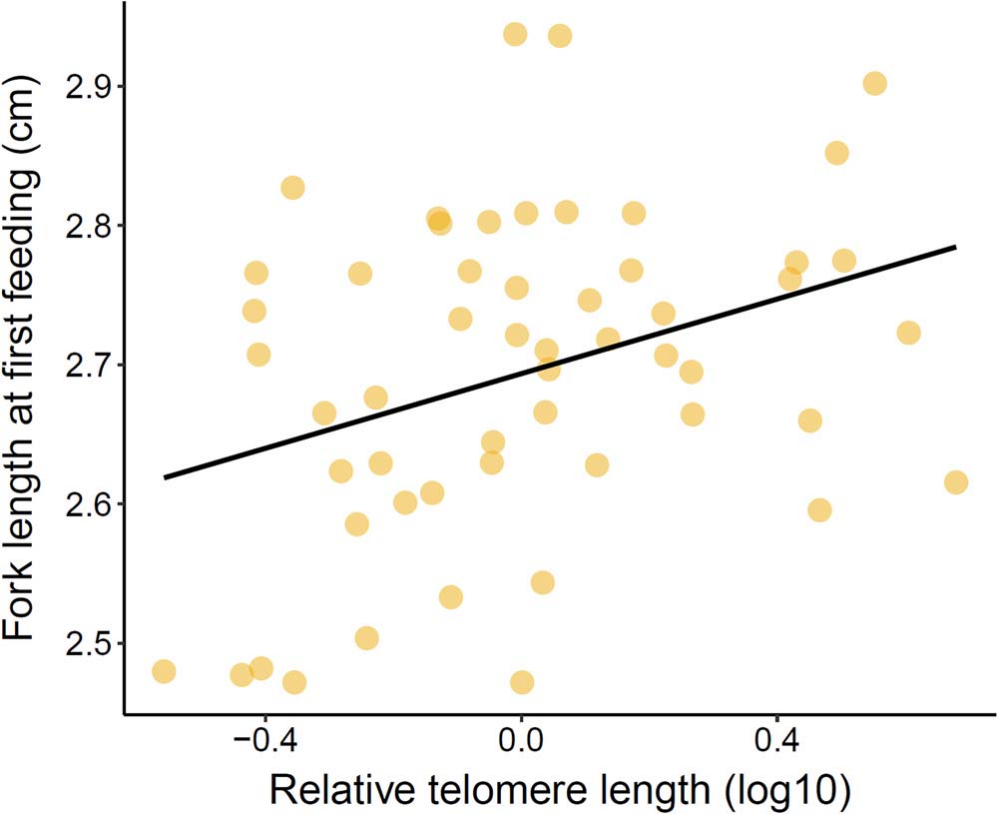
The relationship between family-level relative telomere length at the (earlier) alevin stage of development and family-level offspring fork length at the (later) first feeding stage.

The relative telomere lengths of the salmon offspring were similar among the experimental families, irrespective of the C&R experience of their parents (i.e. which treatment group the parent had been assigned to and whether it was the mother or the father that had undergone the manipulation; Table 1). There was also no link between family-level telomere length (based on the mean of the 3 measured offspring per family) and the overall mortality rate of a given family (Table 2). However, there was a significant link between family-level telomere length and family-level body length (LM: F_1_ = 6.94, *P* = 0.011; Table 2); families with longer mean telomere lengths at the alevin stage were also more likely to be longer in fork length at the first feeding stage (Fig. 2).

**Table 1:** Summary of the linear mixed-effect model explaining variation in offspring RTL in relation to the C&R experimental treatments.

Explanatory variable	Estimate	SE	df	*t*	*P*
Intercept	−0.038	0.088	49.898	−0.436	0.665
Parental stress category B	0.070	0.112	50.039	0.625	0.535
Parental stress category C	−0.004	0.112	49.884	−0.034	0.973
Parental stress category D	−0.161	0.112	49.884	−1.439	0.157
Sex of experimental parent	0.133	0.078	49.969	1.697	0.096

Notes: family ID was included as a random factor to account for non-independence between siblings. The family variance was 0.075 (SD 0.275) and the residual variance was 0.026 (SD 0.161).

**Table 2:** Summary of the general linear model explaining variation in family-level offspring RTL in relation to family-level body length and offspring mortality rate.

Explanatory variable	Estimate	SE	*t*	*P*
Intercept	−2.661	1.056	−2.519	0.015
Fork length at first feeding	0.980	0.382	2.567	0.013
Offspring mortality rate	0.001	0.002	0.509	0.613

Notes: See Section 2.4 for a fuller overview of the model.

## Discussion

The relative telomere lengths of the experimental salmon offspring were unrelated to the C&R experience of their parents. This lack of impact on offspring telomeres is somewhat surprising given the spectrum of negative effects that have already been associated with C&R angling (Cook *et al.,* 2015; Keretz *et al.,* 2018; Twardek *et al.,* 2018; Smukall *et al.,* 2019; Papatheodoulou *et al.,* 2022). However, it remains unclear whether this is due to the absence of an effect, or whether any potential effects are being buffered e.g. via telomere elongation mechanisms.

At any given time, an individual’s telomere length is the product of its initial telomere length and the degree of telomere change it has experienced since then (be that shortening or lengthening). While initial telomere length is often discussed in a post-embryonic context, i.e. on the day of birth, it can also be considered in an embryonic context as the telomere length that is initially inherited via the gametic cells. Supposing that a parent salmon has endured a stressful C&R experience to the extent that it has altered the telomere length of the gametes, offspring may then go on to inherit this telomere length by direct inheritance and thus share the burden of the stressful C&R event. Moreover, offspring may inherit the telomere dynamics of their parents via epigenetic regulation and/or parental effects e.g. passed from the mother via variation in stress hormone levels in oocyte cytoplasm (Blasco, 2007; Entringer *et al.,* 2018). However, reported heritabilities for telomere length vary widely (Dugdale and Richardson, 2018; Chik *et al.,* 2022), partly because parent and offspring telomere lengths are not always measured at the same developmental stage and telomere lengths can change rapidly in early life. For example, germ cells often express high levels of telomerase (Entringer *et al.,* 2018), and there have been various reports of telomere elongation during the early stages of embryonic development, which may serve to reprogramme offspring telomeres to some degree (Schaetzlein *et al.,* 2004; Liu *et al.,* 2007; Kalmbach *et al.,* 2014; McLennan *et al.,* 2018b), which may potentially also buffer any stress-induced changes in telomere length. Moreover, Noguera *et al.* (2022) found that when corticosterone was experimentally administered to the eggs of yellow-legged gulls *Larus michahellis*, the arising offspring had a higher telomerase expression during embryonic development, had longer telomeres at hatching and an associated higher survival probability. Therefore, adverse maternal conditions may even confer benefits to offspring via the adaptive adjustment of their telomere dynamics.

It is also worth noting that, while telomeres in endothermic somatic tissues tend to shorten throughout post-natal life, the telomere dynamics of ectotherms covary with age in less predictable patterns; with various reports of shortening, lengthening, or even a combination of both as an individual ages (Rollings *et al.,* 2014; Ujvari *et al.,* 2017). This may be linked to the fact that most ectotherms are capable of growing throughout life, and may thus maintain telomere elongation mechanisms later into their life as a way of balancing this sustained proliferative capacity (Gomes *et al.,* 2010). This is particularly relevant to Atlantic salmon since their growth patterns differ from many other species in that most of their adult body mass (up to 99%) is attained later in life when they have migrated out into the ocean.

Aside from the experimental manipulation, we found no evidence of a link between family-level telomere dynamics and the total mortality rate of the families (summed over the first three months post feeding). However, we did identify a positive relationship between body size and telomere length, with families that had longer mean telomere lengths at the larval alevin stage also having longer fork lengths at the first feeding fry stage. Rate of growth has been negatively linked to telomere dynamics among numerous different taxa (for review see Monaghan and Ozanne, 2018), since a faster rate of growth may be associated with an increased number of cell divisions and/or level of oxidative stress (Arendt, 2007; Kim *et al.,* 2011; Smith *et al.,* 2016), both of which accelerate the rate of telomere shortening. We have previously identified similar negative effects of growth on juvenile salmon telomeres, both in the lab (McLennan *et al.,* 2018a) and in the field (McLennan *et al.,* 2016; McLennan *et al.,* 2021); but see also McLennan *et al.* (2017). However, as previously mentioned, telomere length can be extremely dynamic during the embryonic and larval stages of salmon development and has also been shown to significantly increase during these periods (McLennan *et al.,* 2018b). Moreover, the rate of embryonic telomerase expression can increase as a result of stress (Noguera *et al.,* 2022) and telomerase expression in fish has been previously shown to correlate positively with cell proliferation rate (Yap *et al.,* 2005; Peterson *et al.,* 2015), thus highlighting the ways in which telomere dynamics may also correlate positively with an individual’s rate of growth.

In summary, we have found no evidence that the telomere lengths of salmon offspring were affected by their parents having experienced stress by simulating catch and release angling. This is reassuring in the sense that it suggests minimal impact of C&R angling close to the time of spawning on the biological state and rates of ageing in the next generation. However, this is only a small part of a complicated picture and as previously mentioned, there is evidence that C&R angling can have other negative effects on the reproductive success of fish such as salmon (Richard *et al.,* 2013; Papatheodoulou *et al.,* 2022). Therefore, we should continue aiming to refine these methods in order to minimize their impacts on fish populations, and thus allow a more considered balance between the welfare of the fish, whilst preserving the socio-economic interests of recreational angling.

## Funding

This work was supported by a Marine Scotland PhD scholarship awarded to M.P., while N.B.M. and D.M. were funded by ERC Advanced Grant 834653.

## Authors’ Contributions

E.D., M.P., N.B.M and D.M. conceived the ideas and designed the methodology; E.D. and M.P. collected the data; and E.D. and D.M analysed the data and led the writing of the manuscript. All authors contributed critically to the drafts and gave final approval for publication.

## Acknowledgements

We thank the staff of the Cromarty Firth Fishery Board, in particular Edward Rush, for their logistical assistance in this experiment; and Shaun Killen, Libor Závorka and Barbara Koeck for help with running the C&R simulations. We also thank Pat Monaghan and Winnie Boner for the use of their molecular laboratory, and Jack Marston and two referees for comments on an earlier version of the manuscript.

## Data Accesibility

Data are available via the Dryad Digital Repository https://doi.org/10.5061/dryad.9s4mw6mn5

## Conflicts of Interest

The authors have no conflicts to declare.
